# Neurons differentially upregulate type 2 immune cytokines and interleukin-4 receptor subtypes during neuroinflammation

**DOI:** 10.1186/s40478-026-02229-7

**Published:** 2026-02-12

**Authors:** Micaela Domingues, Yvonne Gärtner, Nicholas Hanuscheck, Samantha Schmaul, Frauke Zipp, Christina Francisca Vogelaar

**Affiliations:** Department of Neurology, Research Center for Immunotherapy (FZI) and Focus Program Translational Neuroscience (FTN), Rhine-Main Neuroscience Network (rmn 2 ), University Medical Center of the Johannes Gutenberg University Mainz, Langenbeckstr. 1, 55131 Mainz, Germany

**Keywords:** Central nervous system, Neuronal, Type 2 immune cytokines, Interleukin-4 receptor, Interleukin-13 receptor alpha 1, Interleukin-2 receptor gamma, Experimental autoimmune encephalomyelitis, Neuroinflammation

## Abstract

Although numerous studies demonstrate beneficial effects of exogenous interleukin-4 (IL-4) in different central nervous system (CNS) disease models, little is known about the expression of IL-4 and its receptor (IL-4R) system in neurons. We previously showed that the beneficial effects of exogenous IL-4 in neuroinflammation depend on functional neuronal IL-4Rα. Here, we demonstrate neuronal expression of IL-4Rα, the receptor co-chains IL-2Rγ (IL-4R type I) and IL-13Rα1 (IL-4R type II), as well as the type 2 immune cytokines IL-4 and IL-13 throughout the brain and spinal cord of healthy mice and of mice subjected to experimental autoimmune encephalomyelitis (EAE). Surprisingly, we observed differential expression of the receptor chains in different regions of the CNS, with forebrain neurons expressing mainly IL-4R type II and spinal cord neurons showing prominent IL-2Rγ signals (IL-4R type I), the latter mainly known in immune cells. Moreover, we found that the regulation of the system was mainly driven by the increase of IL-4Rα-expressing neurons at the peak of neuroinflammation, whereas the co-chains are downregulated. Additionally, we showed neuronal expression of IL-4 and IL-13 throughout the CNS and observed upregulation of these cytokines during neuroinflammation. Our data indicate that neurons upregulate both IL-4Rα and its ligands during neuroinflammation.

**Supplementary Information:**

The online version contains supplementary material available at 10.1186/s40478-026-02229-7.

## Introduction

Interleukin-4 (IL-4) is a type 2 immune cytokine mainly involved in fighting parasite infections, promoting wound healing, and suppressing pro-inflammatory cytokines [1, 2]. Many cell types in and outside of the immune system are responsive to IL-4, and to the closely related cytokine IL-13 [3]. Both molecules mediate their effects upon binding to the heterodimeric IL-4 receptor (IL-4R) consisting of the subunits IL-4Rα and IL13Rα1, to form the type II IL-4R complex. IL-4Rα can also heterodimerize with the IL-2Rγ chain, which is shared by many other cytokines, such as IL-2, IL-7, IL-15 and IL-21. This heterodimeric complex is called IL-4 receptor type I, is bound by IL-4 but not IL-13, and is mainly expressed by lymphocytes and myeloid cells. Type II IL-4R is more widely expressed in both hematopoietic (e.g., myeloid cells) and non-hematopoietic cells, e.g., endothelial cells, astrocytes and fibroblasts [3–5]. We and others showed that neurons in the cortex and hippocampus of the central nervous system (CNS) express IL-4Rα and play direct roles in neuronal functions, such as outgrowth and synaptic transmission [6–10].

In the past few decades, IL-4 emerged as a neuroprotective and/or –regenerative treatment in experimental disorders of the CNS, such as stroke, traumatic injury and neuroinflammation [11]. Most of these studies focused on microglial effects and did not study neuronal receptor expression nor employ conditional knock outs to prove a causal relation between microglial phenotypes and the functional effects of IL-4. We have proven in neuron-specific IL-4Rα-deficient mice that the beneficial effects of exogenous IL-4 in neuroinflammation depend on neuronal IL-4Rα expression [7]. We observed a direct and fast signaling pathway in neurons, leading to regenerative outgrowth both in vitro and in vivo. Moreover, it has been shown that mice lacking IL-4 and IL-13 display impaired learning behavior [12, 13]. We observed IL-4Rα expression and signaling in the synapse and showed that neuron-specific IL-4Rα mice display alterations in synaptic vesicle recruitment and neuronal hyperactivity, accompanied by hyperactive exploratory behavior and an impairment of fear learning [9]. Neuronal IL-4 plays a role in engram formation during learning, and induces transcriptional changes in murine and human neurons [9, 10]. Interestingly, a recent paper by Li et al. described synaptic expression of IL-13 and IL13Rα1, and activation of downstream molecules involved in synaptic plasticity, thus, IL-4R type II would be the most likely synaptic IL-4R [14].

Despite the prominent role for IL-4 in the nervous system, it is not entirely clear how widespread the neuronal expression of IL-4R in the CNS is. Focusing on the main IL-4Rα chain, we and others showed that IL-4Rα is expressed in neurons in the cerebral cortex and hippocampus [7, 9, 10] but so far, details on other brain regions, and especially on the expression of the co-chains are lacking. Now, we characterized the CNS expression of IL-4Rα, IL-13Rα1, and IL2Rγ, as well as both type 2 cytokines, throughout the brain and spinal cord of healthy mice, and in mice that underwent experimental autoimmune encephalomyelitis (EAE). Despite reports on single-cell RNA sequencing that tend to underestimate the neuronal expression of IL-4R chains, we report expression of IL-4R subtypes in neurons throughout the CNS. We observed interesting differences between brain and spinal cord, and selective changes in expression upon neuroinflammation, supporting a prominent direct function in neurons, which is in line with the therapeutic potential of IL-4.

## Materials and methods

### Animals

C57BL/6 mice were purchased from Envigo. Il2rg KO mice (IMSR_JAX:003174) were purchased from Jackson Laboratories. Animal procedures were performed in accordance with the European Union normative for care and use of experimental animals, conducted according to the German Animal Protection Law and approved by the appropriate state committees for animal welfare (Landesuntersuchungsamt Rheinland-Pfalz, approval G21-1-031).

### EAE

Active EAE was induced in C57BL/6 mice as previously described [7] by subcutaneous injection of 200 µg myelin oligodendrocyte protein 35–55 (MOG_35 − 55_) in combination with complete Freund´s adjuvant (CFA, enriched in mycobacterium tuberculosis H37Ra, 400 µg) followed by an intraperitoneal (i.p.) pertussis toxin (PTX, 200 ng in PBS) injection on day (d)0 and d2. Mice were scored for clinical symptoms daily by a blinded investigator, and signs of EAE were translated into clinical score as follows: 0, no detectable signs of EAE; 0.5, tail weakness; 1, complete tail paralysis; 2, partial hind limb paralysis or ataxia; 2.5, unilateral complete hind limb paralysis; 3, complete bilateral hind limb paralysis or severe ataxia; 3.5, complete hind limb paralysis and partial forelimb paralysis; 4, total paralysis of forelimbs and hind limbs (mice with a score above 3.5 to be killed); 5, death; mice were sacrificed for further analysis at disease peak (score 2.5-3). For RNA isolation and qPCR, animals were transcardially perfused with PBS and the thoracic region of spinal cord was dissected, snap-frozen on dry ice, and stored at -80 °C. For RNAscope, animals were perfused with 4% PFA, spinal cords and brains were post-fixed in 4% PFA and then further processed for sectioning with a vibratome (free-floating in PBS) or cryostat (sucrose preservation and freezing in Tissue Tek on dry ice, stored at -80 °C).

### Cytokine injection

C57BL/6 mice were anaesthetized with inhalation narcosis (isoflurane/O_2_) und analgesia with Rimadyl (Zoetis, 4 µg/g). A hole was drilled in the skull 1.0 mm lateral to the midline at Bregma 1.0 to stereotactically inject 2 µl of interferon gamma (IFNγ, Sigma, 62.5 ng) and tumor necrosis factor alpha (TNFα, Sigma, 62.5 ng) (IFN/TNF), or vehicle (PBS), at 1.05 mm depth. Animals were allowed to recover for 2 days before perfusion with 4% PFA and isolation of the brain for vibratome sectioning.

### Tissue punches

Tissue punches were obtained from the brains of female C57BL/6 mice subjected to transcardial perfusion with PBS, in order to wash out blood cells. The brains were placed in an ice-cold 1 mm spaced brain slicer matrix (Zivic Instruments) with the ventral side facing up. Razor blades were placed at 1 mm distances, in the antero-posterior direction starting at the level of the optic chiasm. The slices were then rapidly transferred to dry ice and tissue biopsies were collected from different brain regions with disposable biopsy punches (pfm medical). Bordering structures, like meninges and choroid plexus were avoided to ensure analysis of the parenchyma only. Biopsies were frozen in Eppendorf vials on dry ice, and stored at -80 °C, prior to RNA isolation.

### RNA isolation

RNA was isolated from tissue biopsies using the RNeasy Micro Kit (Qiagen) according to the manufacturer’s instructions. The eluted samples were treated with RNase-free DNase I (Roche) and the RNeasy MinElute RNA cleanup kit (QIAGEN) was used for further RNA purification. RNA isolation from spinal cord tissue was performed with TRIzol^®^ reagent (Life Sciences), followed by the addition of chloroform. Subsequently, samples were centrifuged for 15 min at 12,000 rcf at 4 °C and the upper/clear phase was collected, followed by isopropanol addition and 1 h centrifugation at maximal speed at 4 °C. The pellets were resuspended with 70% ethanol, followed by 10 min centrifugation at maximal speed and the pellets were eluted in 50 µl RNase-free MilliQ water. For further total RNA purification, samples were treated with RNase-free DNase I (Roche) and RNeasy MinElute RNA cleanup kit (QIAGEN) according to the manufacturer’s instructions.

### RT-qPCR

RT-qPCR was performed as previously described [9]. Briefly, total RNA was supplemented with random hexamer primers and oligo(dT), and incubated for 5 min at 65 °C. Reverse transcription was performed with the SuperScript^®^ III First-Strand Synthesis System (Invitrogen) according to manufacturer’s protocol. qPCRs were performed using the CFX96™ Real-Time PCR Detection System (Biorad). Primers are listed in Table 1. Ribosomal protein S29 (*rps29*) was used as housekeeping gene.

**Table 1:** Primers used for qPCR

Gene	forward	Reverse	Accession nr. / amplicon
*il4*	TCATCCTGCTCTTCTTTCTC	TCCTGTGACCTCGTTCAA	NM_021283.2 / 85–185
*il13*	CATCACACAAGACCAGAC	AATCCAGGGCTACACAGA	NM_008355.3 / 194–282
*il4ra*	TGCTGTTGGTGACTGGAT	TGGAAGTGCGGATGTAGT	NM_001008700.3 / 302–379
*il13ra1*	CATTATCCACTTCAATAGC	AAGACCAGCAATAACATA	NM_133990.4 / 1845–1876
*il2rg*	TGTTGGTTGGAACGAATG	TCTCAGTCAGCCCTTTAG	NM_013563.4 / 930–1044
*rps29*	CAAATACGGGCTGAACAT	GTCGCTTAGTCCAACTTAA	NM_009093.3 / 155–235

Purchased from Metabion

### Immunohistochemistry on vibratome sections

Vibratome (HM650V; Thermo Fisher Scientific) sections from brain were permeabilized with 0.2% Triton in PBS for 10 min, followed by 10 min incubation with 50 mM NH_4_Cl solution. After 1 h blocking (1% BSA, 5% normal goat or donkey serum, and 0.2% Triton-X-100) at room temperature (RT), sections were stained at 4 °C with the following primary antibodies, diluted in blocking solution: anti-IL-4Rα-Alexa 647 (1:50, BD Bioscience-564084, 3 d incubation) and anti-IL-13Rα1 (1:50, ABNOVA-PAB18396, 1 d incubation), or anti-CD4-Alexa 647 (1:100, BD Bioscience-557681, 1 d incubation). After washing, incubation with Alexa Fluor-conjugated secondary antibodies (diluted in blocking solution) was performed for 3–4 h at RT in the dark. After washing, counterstaining was performed with anti-NeuN (1:500, Milipore-MAB377) overnight at 4 °C in the dark, followed by washing and secondary antibody incubation for 3–4 h at RT in the dark. After washing, the slices were stained with DAPI for 15 min, washed, transferred onto a slide, dried, and coverslipped with Prolong gold.

### RNAscope

RNAscope in situ hybridization was performed on 20 μm-thick cryostat sections of brain and spinal cord of healthy mice and mice subjected to EAE, or on vibratome sections of the cytokine-injected brains, that were mounted on Superfrost-Plus (Menzel) slides. The Multiplex Fluorescence v2 (323100; Advanced Cell Diagnostics (ACD)) and 4 Plex Ancillary kits (323120; ACD) were used as previously described [9]. Briefly, sections were dehydrated in a series of increasing ethanol concentrations (50, 70, and 100%) and quenched with hydrogen peroxide for 10 min at RT. Antigen retrieval in 1x RNAscope target retrieval solution (ACD) was performed for 5 min at 99 °C, after which sections were incubated with protease III (ACD) for 30 min at 40 °C. Hybridization with target probes (Table 2) (all ACD Biotechne) was performed for 2 h at 40 °C. Subsequent signal amplification and detection was performed according to the manufacturer’s protocol using OPAL 570, OPAL 520, OPAL 620, or OPAL 690 tyramide reagents (NEL811001KT; all Akoya Biosciences) (all 1:500 dilution in TSA buffer). After DAPI counterstaining, sections were mounted using Pro Long Gold Antifade reagent.

**Table 2:** RNAscope target probes

Target	Name	Cat. Number	Accession number / target region
*il4*	Mm-Il4-C1	312741	NM_021283.2 / 2-498
*il13*	Mm-Il13-C1	312291	NM_008355.3 / 20–632
*il4ra*	Mm-Il4ra-C1	520171	NM_001008700.3 / 845–1770
*il13ra1*	Mm-Il13ra1-C3	437541-C3	NM_133990.4 / 249–1326
*il2rg*	Mm-Il2rg-C2	462211-C2	NM_013563.4 / 2-1093
*nefh*	Mm-Nefh-C4	443671-C4	NM_010904.3 / 622–3074
*vglut1*	Mm-Slc17a7-C3	416631-C3	NM_182993.2 / 464–1415
*vgat*	Mm-Slc32a1-C2	319191-C2	NM_009508.2 / 894–2037

Purchased from ACD Biotechne

### Confocal laser scanning microscopy

Images of immunohistochemistry (IHC) and RNAscope stainings were captured using the Leica SP8 confocal laser scanning microscope. The 20x air, 40x, and 63x oil-immersion objectives were used for different regions of the CNS. For RNAscope images, z-stacks with 1-µm step size were acquired for each channel. For IHC images, tile scans were created using the LAS-X software (Leica).

### Post-processing

ImageJ or Adobe Photoshop CS5 were used for processing IHC or RNAscope images. Brightness and contrast were adjusted equally for all images of the same condition. Maximum projections of all channels were generated over the z axis with equal numbers of planes for images between samples.

### Quantification of RNAscope signal

Neurons positive for *il4ra*, *il13ra1* and *il2rg* were quantified using the ImageJ input “Cell counter”. *Nefh*-positive neurons in maximum z-projections of spinal cord, cortex, and nucleus ruber (NR) images were marked and cells that contained two or more puncta of *il4ra*,* il13ra1* or *il2rg* were counted. The receptor-positive cells were normalized to all neurons counted in the image and values of 10–14 images from 2 to 4 mice per group were included in the analysis. For quantification of the *il4* and *il13* signals in sections of the spinal cord, we set a consistent threshold for *il4*,* il13* and *vgat* in all z-projections based on the control images. The total area above the threshold was then measured for *il4* and *il13*, and related to the area of the *vgat* neurons in this image. Six images from 1 to 2 mice per group were used for analysis. In the cytokine-injected mice (4–6 images of 2 mice per group), the *il4ra* signal was thresholded and then related to the number of *nefh*-positive neurons.

### Statistical analysis

Statistical analyses were performed with GraphPad Prism software (version 9 and 10). Appropriate statistical tests were chosen based on the experimental conditions: unpaired T-test for comparisons of two independent groups; one-way ANOVA for comparison of multiple groups, including post-hoc corrections when multiple groups are analyzed with one common control group.

## Results and discussion

### IL-4R type II is expressed in neurons of different brain regions

**Fig. 1:**
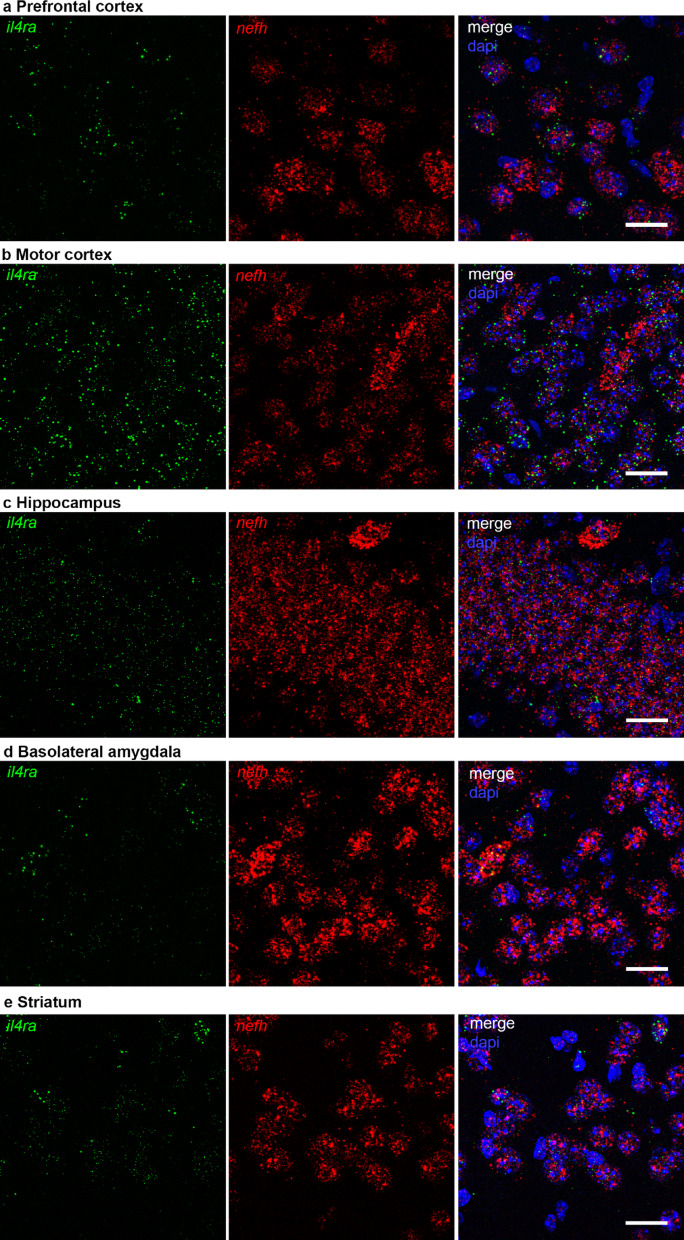
Expression of *il4r* mRNA in neurons in different brain regions. RNAscope for *il4ra* (green) and *nefh* (red), with dapi (blue), in **a** prefrontal cortex, **b** motor cortex, **c** hippocampus, **d** basolateral amygdala, and **e** striatum. Scale bar = 20 μm

**Fig. 2:**
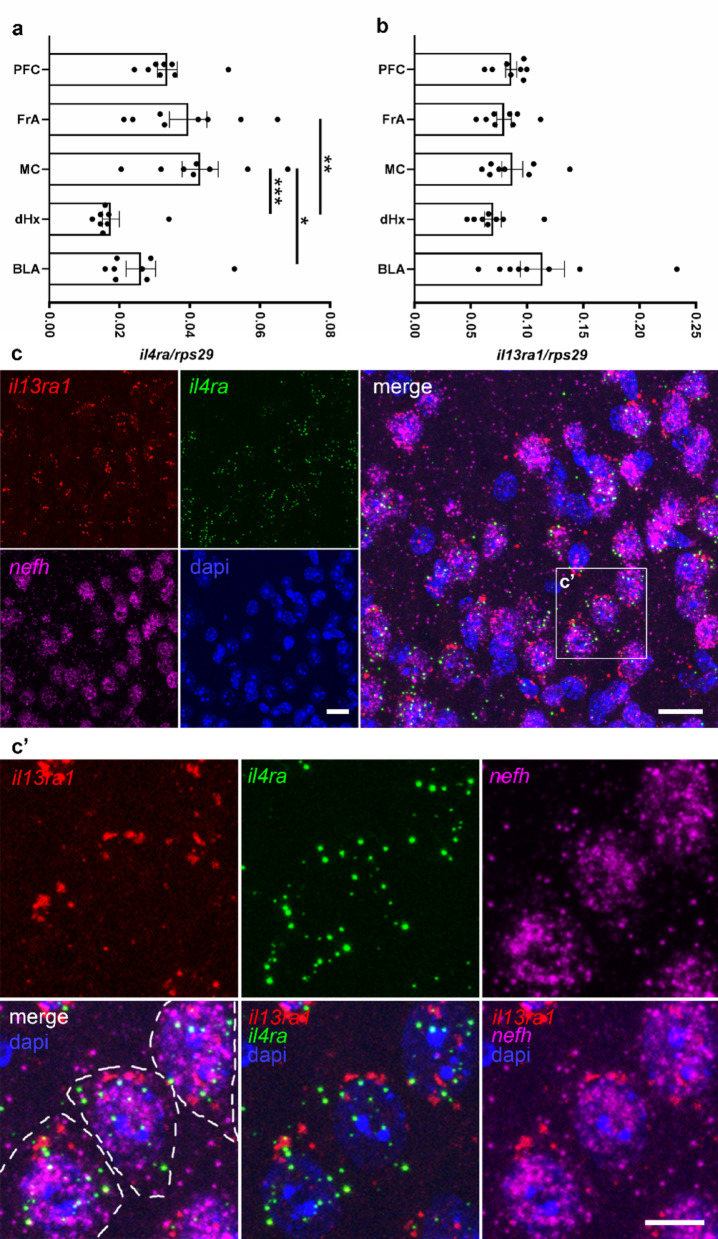
IL-4R type II expression in neurons. RT-qPCR on tissue biopsies revealed expression of **a ***il4ra* and **b ***il13ra1* in different brain areas (n = 2–3 samples per group of 3 independent experiments). **c** RNAscope for *il13ra1* (red), *il4ra* (green) and *nefh* (magenta), with dapi (blue). Boxed area enlarged in **c’** with neurons outlined (white dashes) in merge + dapi. Scale bars = 50 μm (**c**) and 25 μm (**c’**). Statistical analysis: (**a**, **b**) one-way ANOVA with Tukey correction, * *p* < 0.05, ** *p* < 0.01, *** *p* < 0.001. Abbreviations: PFC = prefrontal cortex, FRA = frontal association cortex, MC = motor cortex, dHx = dorsal hippocampus, BLA = basolateral amygdala

Based on our previous findings demonstrating neuronal expression of IL-4Rα in the cortex and hippocampus of healthy mice [9], the current study investigates the expression patterns of IL-4Rα in conjunction with IL-13Rα1 across various brain regions. Immunofluorescence microscopy provided detailed insights into the spatial distribution of these receptor subtypes under basal conditions. First, we confirmed *il4ra* mRNA expression throughout the brain via RNAscope. *Il4ra* mRNA was found to be co-expressed with the neuronal marker *neurofilament h* (*nefh*) mRNA in, among others, prefrontal cortex (PFC), motor cortex (MC), hippocampus (Hx), basolateral amygdala (BLA) and striatum (Fig. 1). This was confirmed with RT-qPCR on tissue biopsies of several regions, in which we also observed expression of *il13ra1*. Interestingly, in contrast to *il13ra1*, which was evenly distributed, *il4ra* expression levels were significantly different between brain regions, with cortical areas expressing higher levels than subcortical regions (Fig. 2a-b). This indicates that IL-4R functions in different brain regions are regulated by expression levels of IL-4Rα. We confirmed neuronal co-expression of *il4ra* and *il13ra1* via RNAscope for both receptor chains in combination with *nefh* (Fig. 2c-c’). Compared to *nefh* mRNA, which is distributed throughout the neuronal cell bodies, *il4ra* and *il13ra1* mRNA were less abundant, reflected by their appearance as puncta in the *nefh*-filled area. This is not surprising, since IL-4Rα is known to be expressed in notoriously low copy numbers even in immune cells [15, 16]. This observation also explains why single-cell RNAseq analysis in the brain showed *il4ra* expression in very few neurons [10, 17]. The absence of detection of a transcript does not necessarily indicate that it is absent in the cells, since the sequencing depth of single-cell RNAseq may not be sufficient to detect low abundant proteins [18] (personal communication with Peter Lönnerberg). In fact, by using a less stringent threshold for expression, the mousebrain.org scRNAseq database [17] shows that most CNS neurons express at least two receptor chains (supplemental Fig. 1a). Moreover, variations in mRNA stability or translation efficacy may greatly influence expression at the protein level. It should also be noted that receptors are at the beginning of the signaling cascade they initiate. Consequently, they are generally expressed at low abundance. Even among IL-4-responsive immune cells, such as B, T, and myeloid cells, IL-4Rα is expressed at very low copy numbers [15, 19, 20]. Despite of these low levels of IL-4Rα, IL-4 and IL-13 are known to act on many cells, including neurons [3, 7, 9].

In order to investigate whether the direct IL-4R downstream signaling molecules Jak1-3, as well as Tyk2, are expressed in neurons, we again turned to the mousebrain.org scRNAseq database. *Jak1*, which associates to the IL-4Rα chain [4, 21], is most prominently expressed, whereas *jak3* (downstream of IL2rγ), *jak2* and *tyk2* (downstream of IL-13Rα1) are less abundant (supplemental Fig. 1b). The expression levels of these signaling molecules were higher than those of the receptor chains (supplemental Fig. 1a), which is expected because Jak molecules are known to be downstream of not only cytokine receptors, but also neuronal receptors for hormones and growth factors [22]. Interestingly, *Jak3* seems less prominent in forebrain neurons than in the mid- and hindbrain (supplemental Fig. 1b, arrows). The presence of these signaling molecules in neurons suggests that the IL-4R system is likely to be functional.

**Fig. 3:**
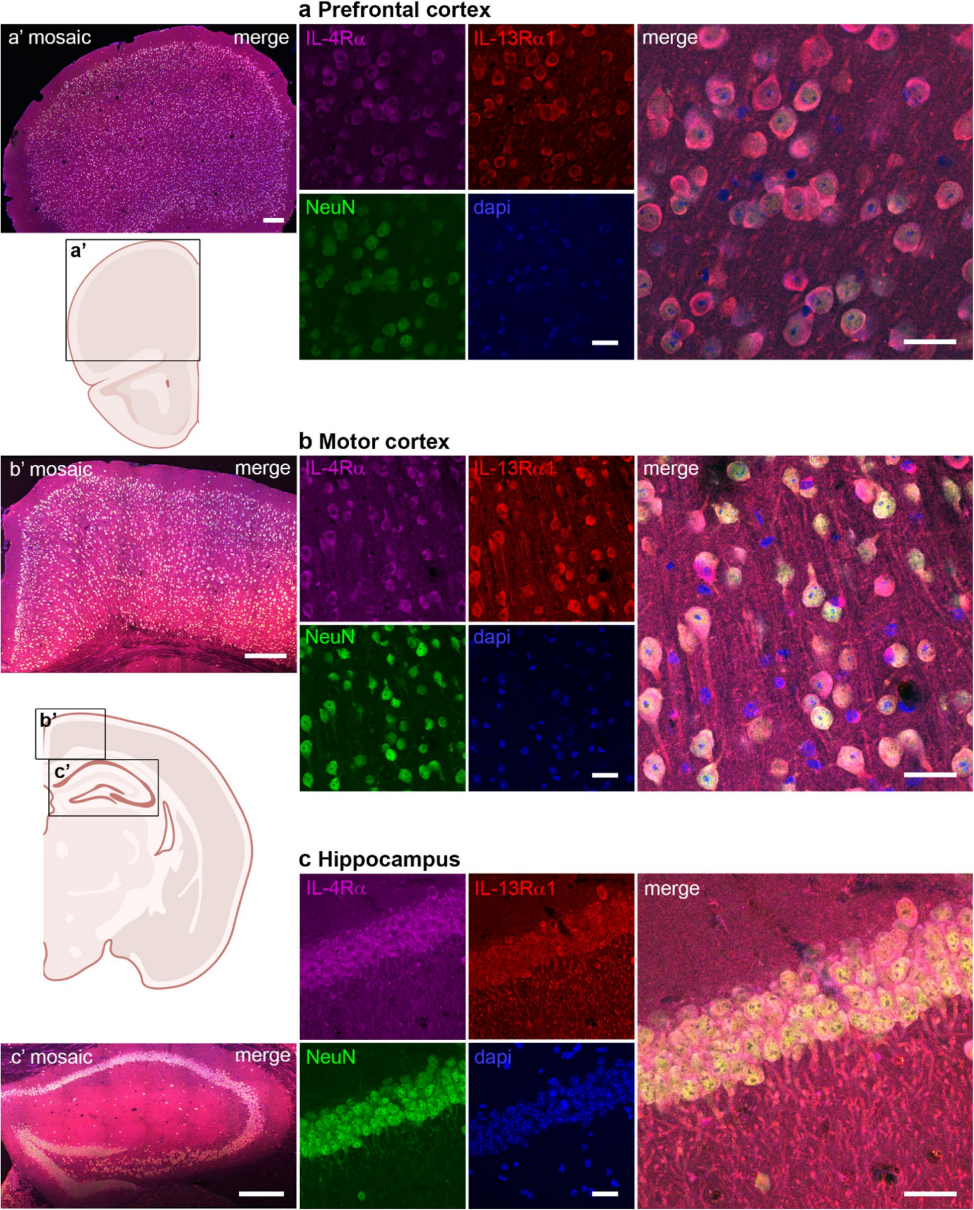

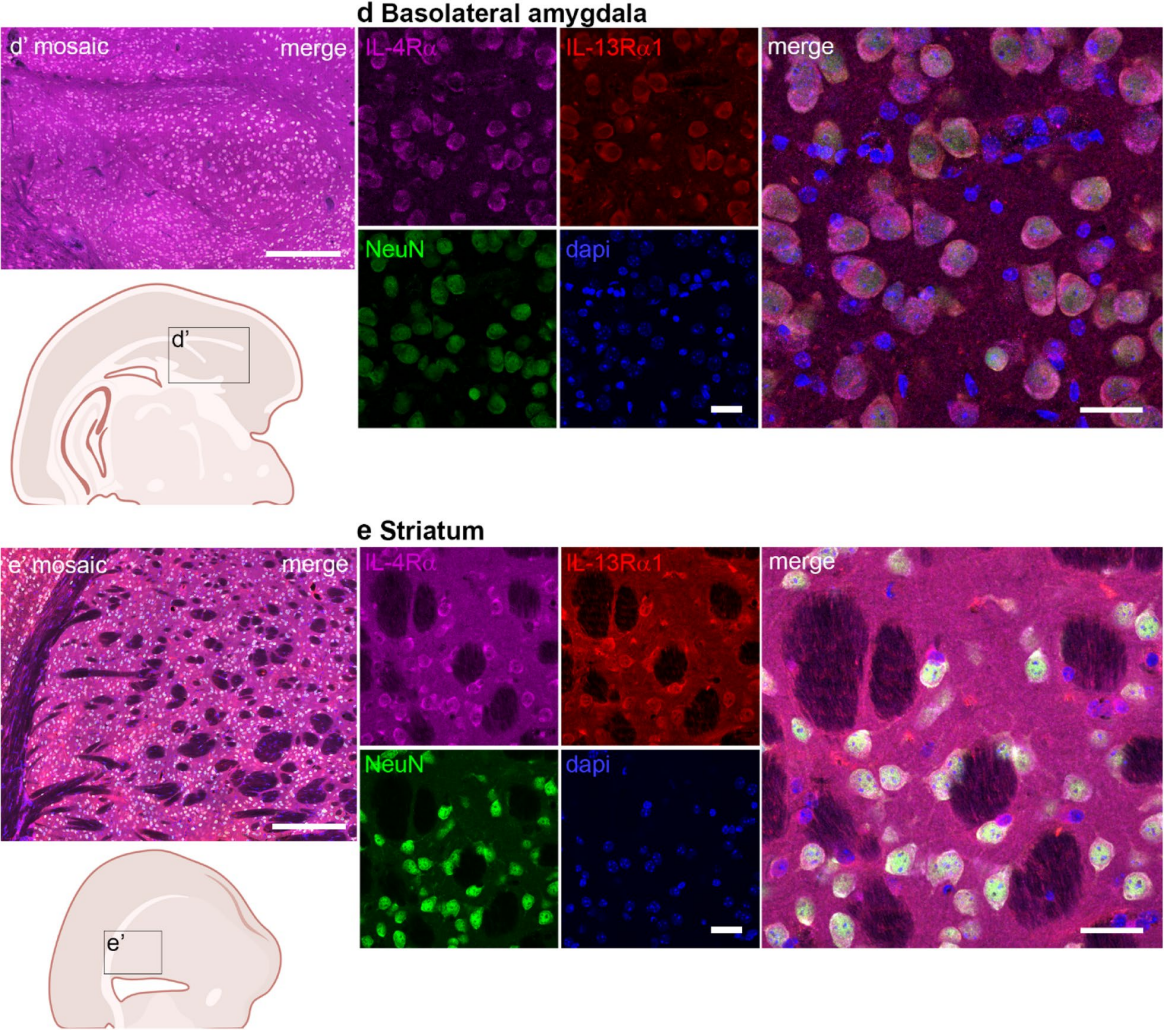
Neuronal co-localization of IL-4Rα and IL13Rα1 protein in different brain regions. Immunohistochemistry for IL-4Rα (magenta), IL-13Rα1 (red), the neuronal marker NeuN (green), and dapi (blue) in coronal sections of brain areas: **a** prefrontal cortex, **b** motor cortex, **c** dorsal hippocampus, **d** basolateral amygdala, and **e** striatum. Scale bar = 20 µm **a’-e’** low resolution mosaic images (scale bar = 250 μm) and approximate location in a schematic overview (created with BioRender) of the sections

On the protein level, we observed via IHC that IL-4Rα and IL-13Rα1 exhibit robust co-expression with the neuronal marker NeuN in several regions, including the prefrontal cortex, motor cortex, hippocampus, amygdala, and striatum (Fig. 3). We additionally confirmed the known IL-4Rα expression by astrocytes, microglia, and oligodendrocytes [11, 23, 24] (supplemental Fig. 2). The differential staining intensities of especially IL-4Rα in different regions (Table 3) again suggests regional differences in IL-4R requirements. The abundance of IL-4Rα and IL13Rα1 did not always match, implying differential regulation or even different functions. Although IL-13Rα1 can only be functional in combination with IL-4Rα in the IL-4R type II complex, it can also dimerize with IL-13Rα2, together forming a decoy receptor [4]. Only a few studies suggest a functional role of IL-13Rα2 in fibrosis [25, 26], but so far, neither expression nor function in neurons has been shown. Consistent with the neuronal circuits known to play a role in fear and anxiety [27, 28], the co-localized expression of IL-4Rα and IL-13Rα1 neurons in prefrontal cortical areas, hippocampus and basolateral amygdala is corroborated by our earlier finding that neuronal IL-4R signaling plays a role in anxiety-like behavior [9].

**Table 3:** IL-4R type II protein in brain regions

Brain region	IL-4Rα	IL-13Rα1
Cortical areas		
Frontal association cortex	+++	++
Motor cortex	++	+++
Prefrontal cortex	++	+++
Visual cortex	+-	+++
***Subcortical areas***		
Basolateral amygdala	+	+
Dorsal hippocampus	+	+
Hypothalamus	++	++
Nucleus accumbens	++	+++
Substantia nigra	+-	+
***Cerebellum***		
Cortex (Purkinje cells)	++	+-

Qualitative analysis of signal intensity of IL-4Rα and IL-13Rα1 in neurons in different brain regionsVisual evaluation: +- = weak, + = moderate, ++ = strong, ++ = very strong

**Fig. 4:**
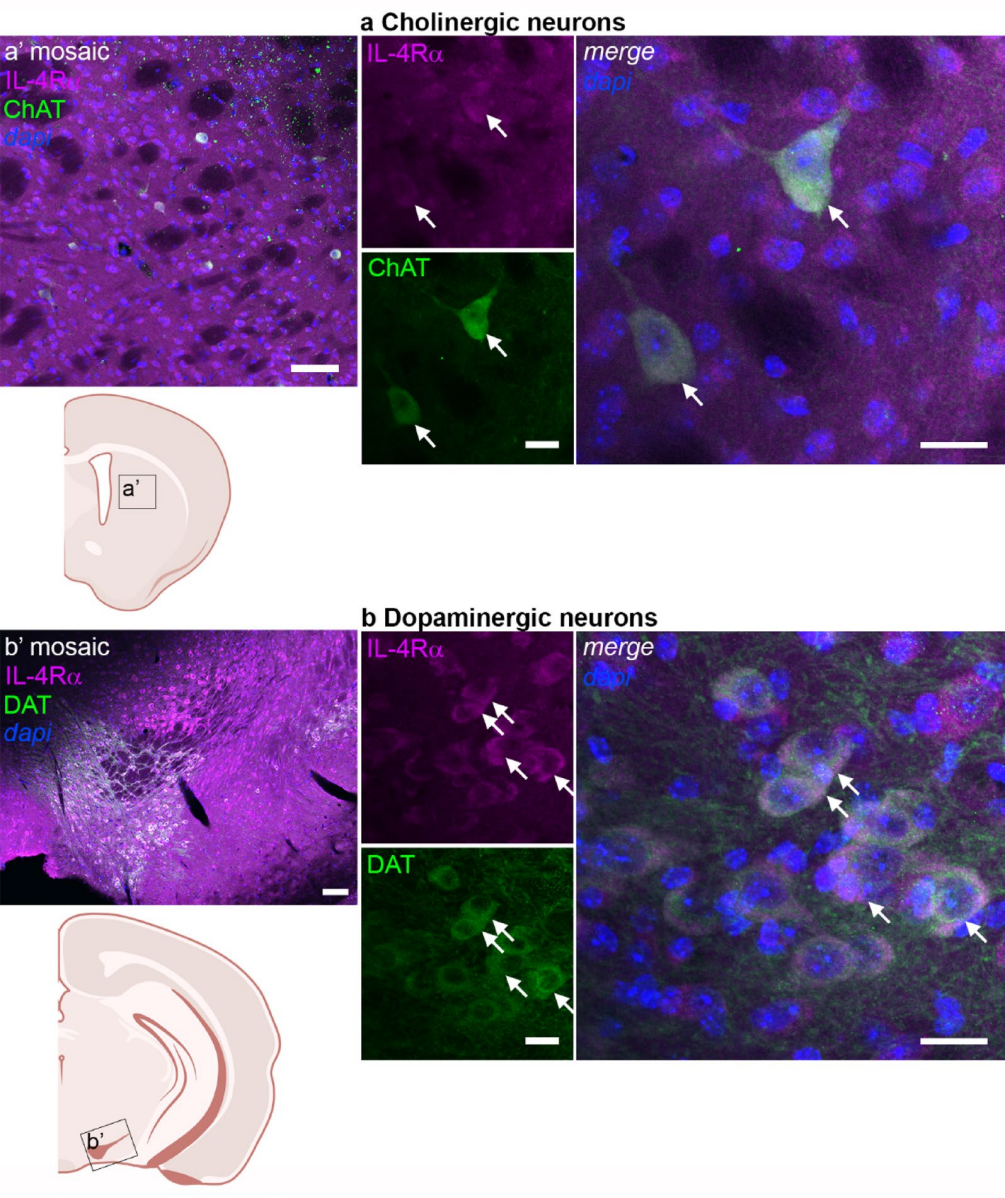
Expression of IL-4Rα in neuronal subtypes.Immunohistochemistry for IL-4Rα (magenta), dapi (blue), and **a** choline acetyltransferase (ChAT, green) in the striatum, and **b** dopamine transporter (DAT, green) in the substantia nigra. Scale bar = 20 µm. Arrows point to cells expressing IL-4Rα and the respective neuronal subtype marker. **a’-b’** Low resolution mosaic images and approximate location (scale bar = 100 μm) in a schematic overview (created with BioRender) of the sections

In addition to our previously published observation of IL-4Rα expression in both excitatory and inhibitory neuronal subtypes [9], we now show that dopaminergic neurons in the substantia nigra, and cholinergic neurons in the striatum express IL-4Rα (Fig. 4a-b). The expression in dopaminergic neurons is in line with reports showing neuroprotective effects of IL-4 on dopaminergic neurons, although so far, research focused on microglia and contradictory effects were shown depending on the model [29, 30]. Interestingly, the loss of IL-13Rα1 results in reduced dopaminergic cell loss, but this was studied in a conventional knockout mouse. Since both microglia and dopaminergic neurons were shown to express IL-13Rα1, indirect or direct effects could not be unraveled [31]. In the striatum, on the one hand, IL-4 was shown to protect from excitotoxic damage [32]. On the other hand, IL-4 aggravates LPS-induced striatal neurodegeneration via oxidative stress [33]. Apparently, IL-4 cannot exert neuroprotective effects when a strong toxin is directly injected into the striatum. Despite these contradictory downstream effects, the main conclusion here is that IL-4R is expressed and functional in the substantia nigra and striatum.

In summary, here we provide comprehensive insights into the regional distribution of IL-4Rα and IL-13Rα1 in the brain under resting conditions, highlighting the potential role of IL-4R type II in neuronal homeostasis within distinct brain regions.

### *Il4* and *Il13* are expressed by neurons in the murine brain

**Fig. 5:**
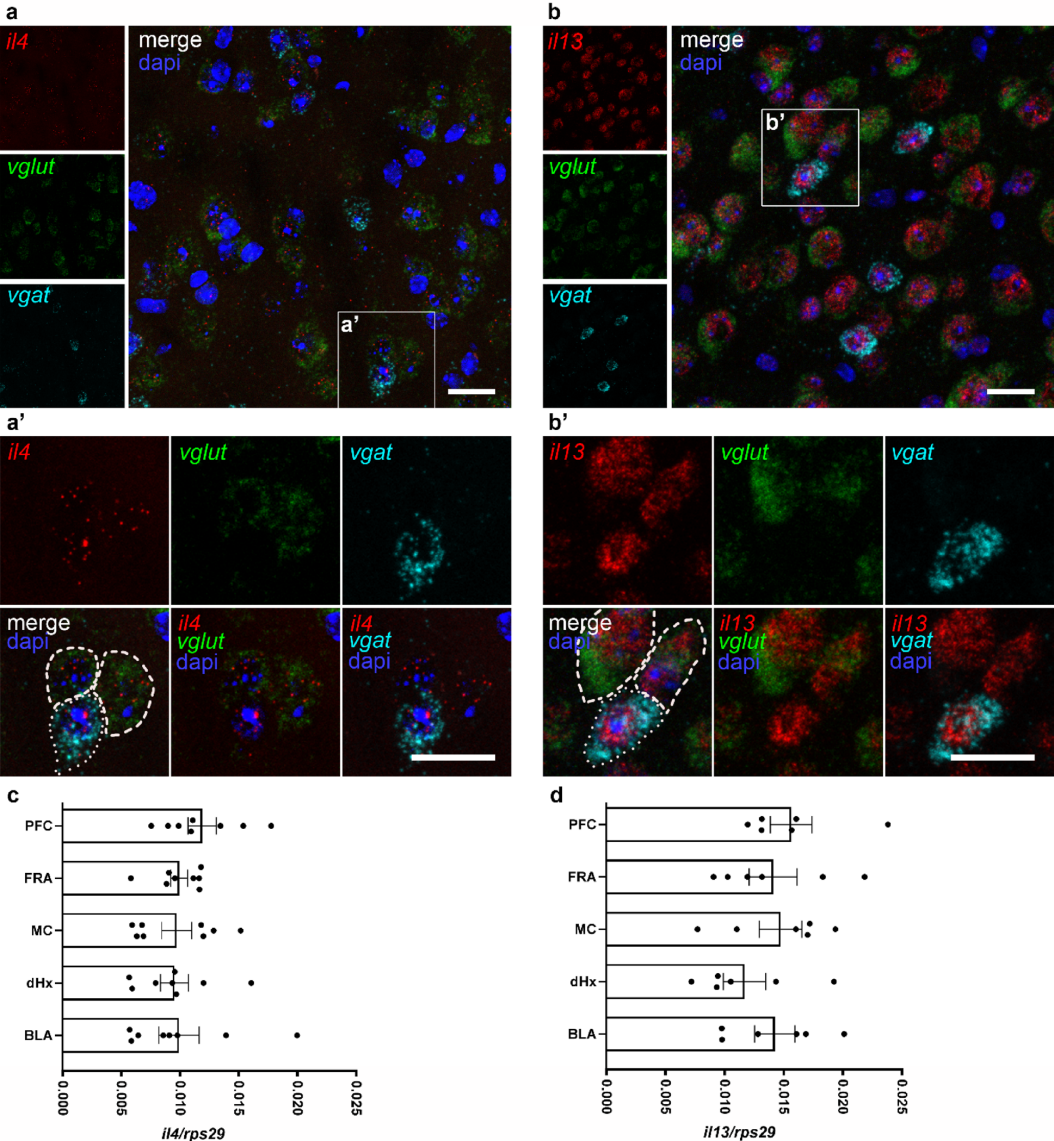
*il4 *and *il13*mRNA expression in the healthy brain. RNAscope for **a-a’ ***il4* (red), and **b-b’ ***il13* (red), with dapi (blue), and markers *vglut* (green) and *vgat* (cyan) for excitatory (dashed lines) and inhibitory (dotted lines) neurons in the motor cortex of adult healthy mice. Scale bar = 50 μm. **c-d** qPCR analysis of *il4* and *il13* in tissue punches of different brain regions, normalized to ribosomal protein *rps29* (*n* = 2–3 samples per group of 3 independent experiments). Statistical analysis (**c**, **d**) one-way ANOVA with Tukey correction,. Abbreviations: PFC = prefrontal cortex, FRA = frontal association cortex, MC = motor cortex, dHx = dorsal hippocampus, BLA = basolateral amygdala

Both IL-4 and IL-13 are able to bind IL-4R type II, therefore, not only the distribution of the receptor chains, but also the expression of these type 2 immune cytokines in the brain has gained importance in recent years. So far, neuronal expression of IL-4 was reported in a murine stroke model [34], but not in the healthy CNS. Both cytokines are implicated in synaptic functions [9, 10, 14], however, most studies focused on IL-4 showing amelioration of disease symptoms [11]. We previously showed that IL-4, but not IL-13, was able to induce axon outgrowth [7]. In the present study, we performed qPCR and RNAscope on brain sections to study differential expression of *il4* and *il13* mRNA in neurons. The advantage of RNAscope is its high sensitivity enabling the detection of single molecules. Here, we demonstrate co-expression of *il4* and *il13* with the neuronal markers *vgat and vglut* (vesicular GABA and glutamate transporter) in excitatory and inhibitory neurons, respectively, in the motor cortex of healthy adult mice (Fig. 5a-b). Analysis of the cytokine levels via qPCR in tissue biopsies of various brain regions showed expression of both cytokines in those regions where the receptors are expressed, with slightly higher levels of *il13* (Fig. 5c-d).

### IL-4R subtypes are upregulated under neuroinflammation in vitro and in vivo

**Fig. 6:**
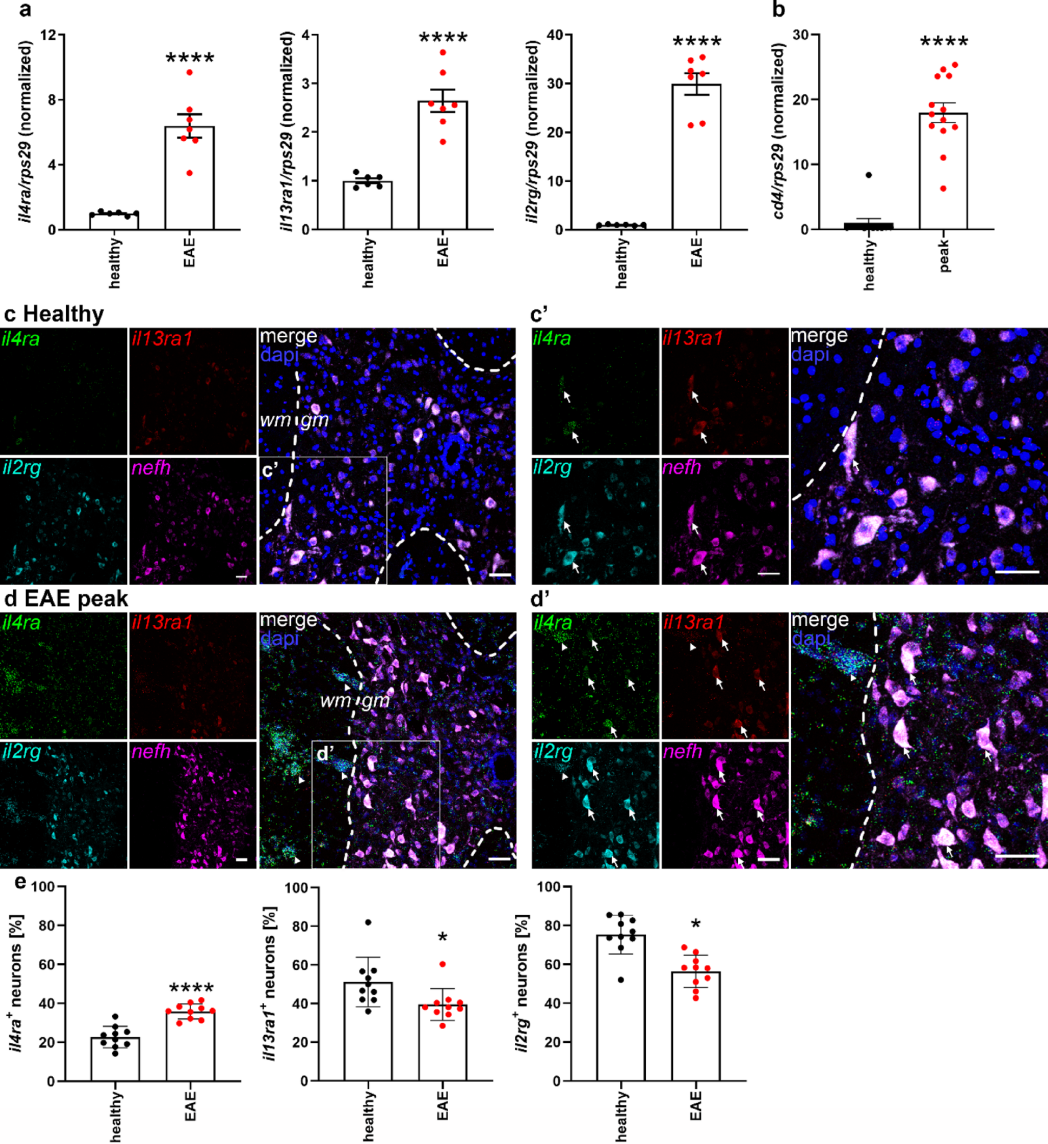
IL-4R chains are upregulated in spinal cord at EAE peak.** a** RT-qPCR for the receptor chains in relation to *rps29* at peak phase of EAE, normalized to healthy controls. **b** RT-qPCR for *cd4* in relation to *rps29* at EAE peak, normalized to healthy controls (n = s.c. of 6–7 animals per group from 2 independent experiments). **c-e** RNAscope for *il4ra* (green), *il13ra1* (red), *il2rg* (cyan), and *nefh* (magenta), with dapi (blue), in spinal cord cross sections from healthy controls and EAE mice at peak. Boxed areas enlarged in **(c-c’)**. Arrows indicate neurons triple-positive for all receptor chains. Arrowheads point to immune cell infiltrates in **(d-d’)**. Scale bar = 50 μm. **e** Quantification of percentage of neurons expressing *il4ra*, *il13ra1*, or *il2rg* (*n* = 10 images from 2–3 mice per group). Statistical analysis: (**a**, **b**, **e**) unpaired T-test. * *p* < 0.05, **** *p* < 0.0001. Abbreviations: gm = grey matter, wm = white matter

Based on our successful treatment of mice subjected to EAE with exogenous IL-4, we now investigated whether inflammation affects the expression of IL-4R subtypes. To this end, we performed RT-qPCR for the receptor chains on the spinal cords of healthy controls and EAE mice (C57BL/6 MOG_35-55_ model) at the disease peak (~ day 12, clinical score 3). We observed a substantial increase in expression of *il4ra*, *il13ra1*, and *il2rg* in EAE animals, as compared to healthy controls (Fig. 6a). Since this increase is likely due to immune cell infiltrates, as illustrated by the increase in *cd4* mRNA expression (Fig. 6b), we then performed RNAscope to obtain cellular resolution. Interestingly, *il4ra* was expressed at relatively low levels contrary to *il13ra1*, expressed at intermediate levels, and *il2rg*, which was highly abundant in *nefh*^+^ neurons in the grey matter (Fig. 6c-c’). At the peak of EAE disease, there was an increase in *il4ra* mRNA puncta, both in neurons in the grey matter (Fig. 6d-d’, arrows) as well as in cells in the white matter, which we identified as immune infiltrates, due to their dense appearance and co-expression of *il2rg* (Fig. 6d-d’, arrowheads). Although lymphocytes mainly express IL-4R type I, granulocytes express both receptor subtypes [1], therefore, *il13ra1* mRNA was observed in the infiltrates as well.

In addition, we quantified the neuronal expression of the receptor chains by counting the fraction of *nefh* neurons positive for either of the chains (Fig. 6e). The neuronal expression of *il2rg* and *il13ra1* slightly decreased at EAE peak compared to healthy controls, so the increase we observed in the qPCR may be mostly due to the presence of infiltrating immune cells. In contrast to the co-receptor chains, the *il4ra* signals increased specifically in neurons (Fig. 6d’ & e). Indeed, in healthy animals, the number of neurons expressing *il4ra* was much lower than those expressing *il13ra1* or *il2rg*, whereas in EAE animals, *il4ra*-positive neurons increased to approximately the same levels as the co-chains. This indicates again that the regulation of the IL-4R complex is performed at the level of the IL-4Rα chain. We observed signals for all chains in non-neuronal cells in the white matter (Fig. 6d-d’, arrowheads). In order to confirm immune cell infiltration, we performed IHC for the T helper cell marker CD4. Infiltration was more prominent in the white matter, with some sparse CD4^+^ cells in the grey matter. We observed immune cell infiltration throughout the spinal cord, independent of the spinal level (supplemental Fig. 3), which was expected since we analyzed samples at EAE peak. Therefore, the thoracic level, where we quantified neuronal receptor chain expression (Fig. 6), should be considered representative for all spinal levels.

**Fig. 7:**
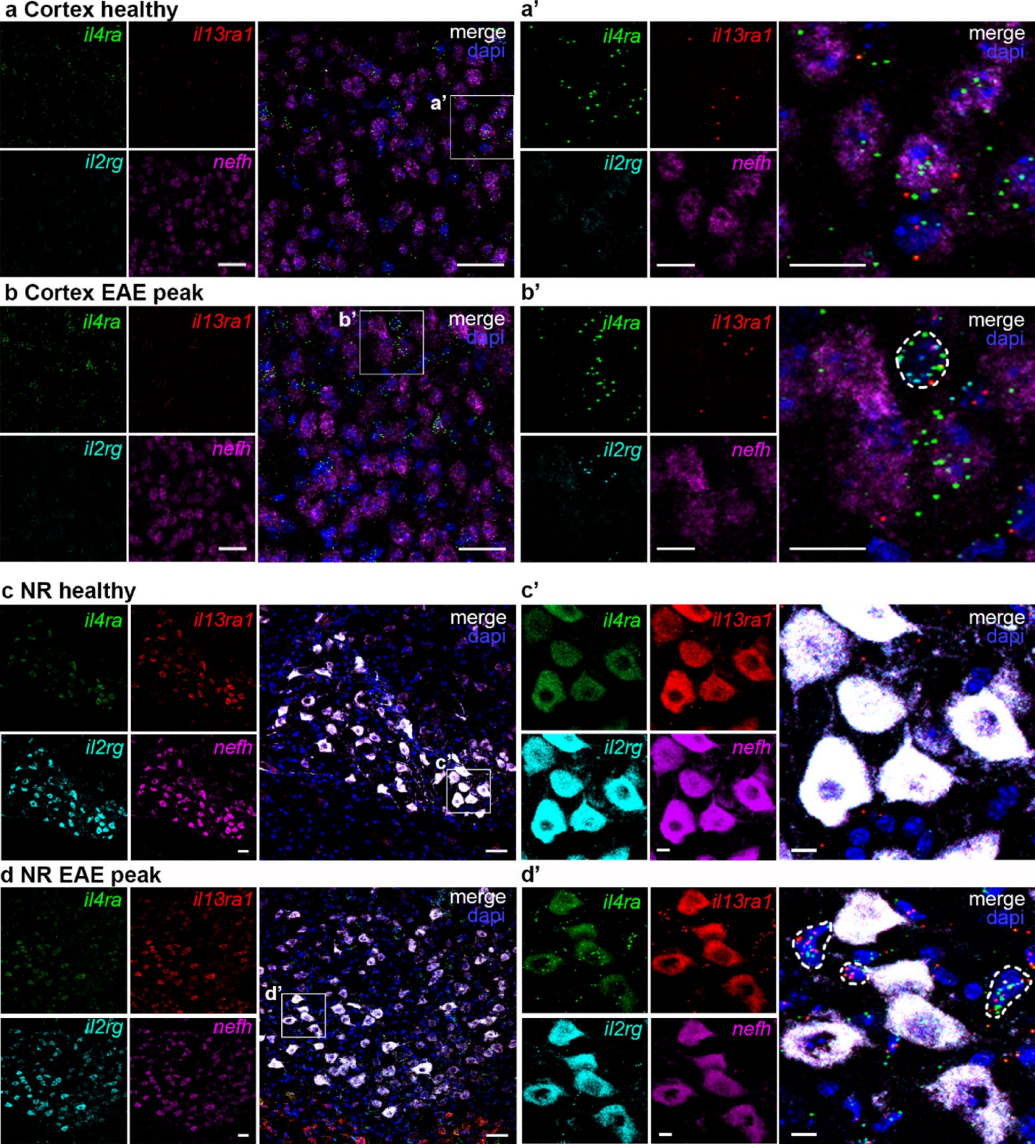
IL-4R chain expression in brain and nucleus ruber during EAE. RNAscope for *il4ra* (green), *il13ra1* (red), *il2rg* (cyan), and *nefh* (magenta), with dapi (blue), in **a**,** b** motor cortex and **c**,** d** nucleus ruber (NR) from healthy controls and EAE mice at disease peak. Scale bar = 50 µm. **a’-d’** Enlargement of boxed areas. Scale bar = 10 μm. Non-neuronal cells marked with dashed lines in EAE peak merge + dapi

Another unexpected finding was the striking difference between spinal cord neurons and cortical neurons with respect to the *il2rg* abundance. Whereas spinal cord neurons showed high expression of *il2rg*, cortical neurons displayed very low basal levels of this chain (Fig. 7a-b). In contrast, neurons in the NR, part of the extrapyramidal motor system in the mesencephalon, also expressed all co-receptor chains, with *il2rg* intensities comparable to those in the spinal cord (Fig. 7c-d). We were unable to confirm expression of IL-2Rγ on the protein level, since available antibodies revealed to be unspecific based on IHC on il2rg knockout brains (data not shown). Experiments on spleen sections confirmed that the specific RNAscope probes were able to detect the receptor chains at their expected levels. The most abundant in receptor chain in the spleen was *il2rg*, which is known to be common to many different interleukin receptors. *Il4ra* was expressed at intermediate levels and *il13ra1* exhibited low abundance. The RNAscope signals for *il2rg* were abolished in il2rg^-/-^ spleens, therefore, we conclude that the probes are specific (supplemental Fig. 4). We have previously established the specificity of the IL-4Rα antibodies and *il4ra* probes by demonstrating that neuronal staining was largely abolished in neuron-specific IL-4Rα-deficient mice [7, 9]. Since IL-4Rα is only functional in complex with one of the co-chains, the co-expression observed with RNAscope, and the colocalization of IL-4Rα and IL13Rα1, strongly suggests that IL-4R type II is the main receptor subtype in the forebrain and hippocampus. In contrast, the more posterior regions, like the nucleus ruber, and the spinal cord, prominently express both co-chains, therefore we conclude that neurons in those regions express both IL-4R type I and II.

We observed RNAscope signals for the different receptor chains in a few non-neuronal cells in the cortex and NR at EAE peak (Fig. 7b’ and d’). Therefore, we performed IHC for CD4 and observed very few immune cells in the cortex, which is in line with the general observation that the forebrain displays no lesions in this model [35]. In the area close to the NR, CD4 expression was equally low. Quantification of the *il4ra* levels in both regions revealed no difference between control and EAE mice (supplemental Fig. 5). Therefore, we conclude that in areas with little infiltration, neurons do not upregulate the receptor.

In order to investigate whether cytokines alone can induce *il4ra* expression, we injected IFNγ and TNFα (IFN/TNF) in the motor cortex of C57BL6 mice. Indeed, we observed an upregulation of *il4ra* RNAscope signals in cortical neurons in areas close to the injection site (supplemental Fig. 6). However, since there was some cell infiltration surrounding the injection tract, we cannot conclusively discriminate between the effects of cytokines and immune cells.

Altogether, in the EAE model, which is known to display pathology primarily in the spinal cord, indeed, the receptor complex is mainly regulated in spinal neurons. The regulation is likely performed through controlling the levels of the main chain *ilr4a*, which is the only chain that was increased at EAE peak. Biologically, this is very plausible, since IL-4Rα is the common chain to both receptor subtypes and therefore represents a limiting factor. Although the neuronal upregulation of *il4ra* correlates with the abundance of immune cell infiltrates, it could also be induced by injecting cytokines into the CNS. We conclude that the increase in *il4ra* levels may be a neuronal response to both cellular as well as soluble components of the immune system.

### IL-4 and IL-13 expression is upregulated during neuroinflammation in spinal cord

**Fig. 8:**
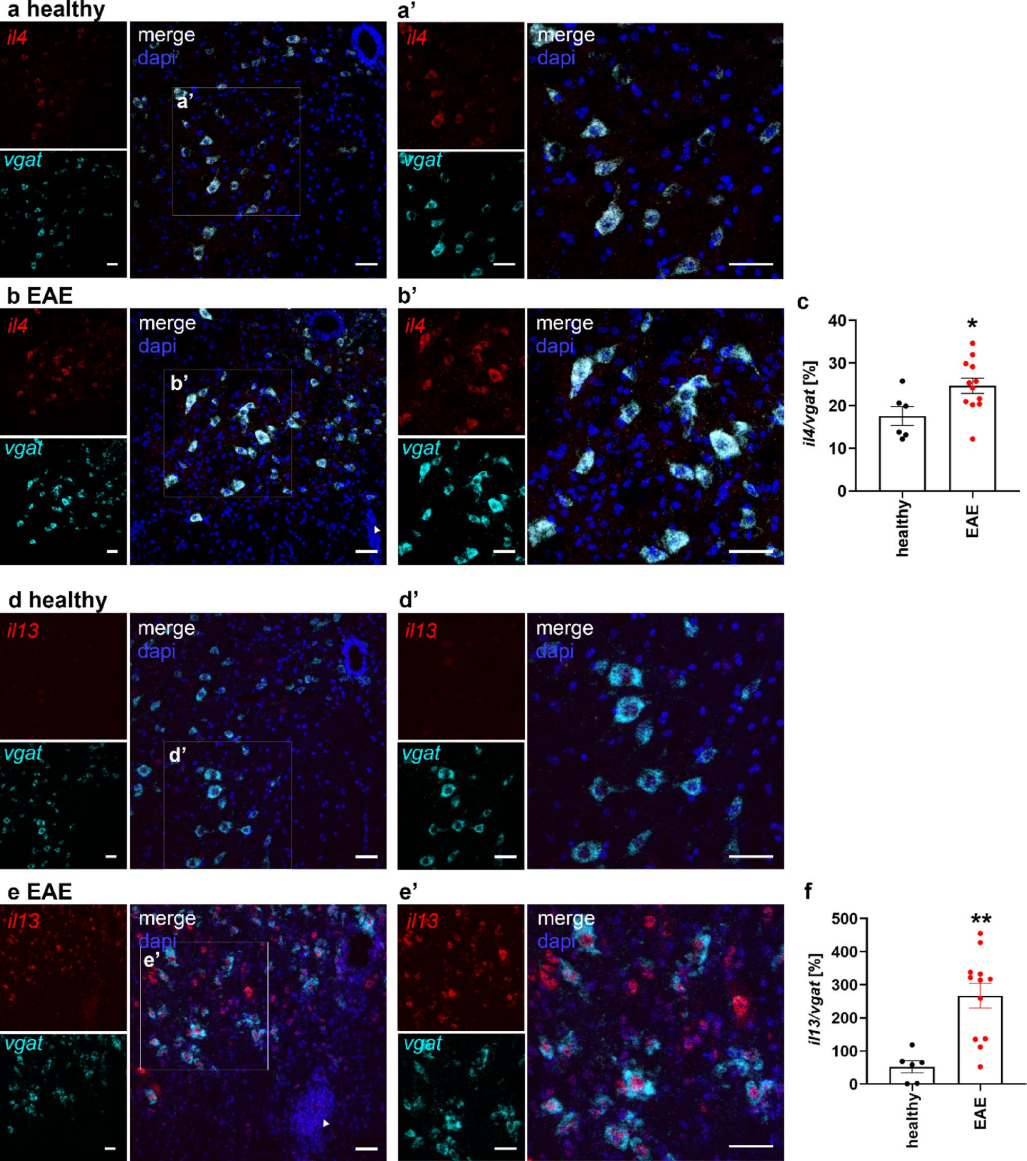
IL-4 and IL-13 are upregulated in spinal cord at EAE peak. RNAscope for **a**, **b** *il4* & **d**, **e** *il13* (both in red) and *vgat* (cyan), with dapi (blue), in spinal cord cross sections from **a**, **d** healthy controls and **b**, **e** EAE mice at disease peak. Boxed areas enlarged in **a´-b´** and **d´-e´**. Scale bar = 50 μm. Quantification of **c ***il4*-positive area and **f** *il13*-positive area normalized to *vgat*-positive area in healthy and EAE spinal cord sections  (6–12 images from 1–2 mice per group). Statistical analysis: (**c**, **f**) unpaired T-test. * *p* < 0.05, **p < 0.001

The neuronal upregulation of IL-4Rα in response to inflammation at EAE peak in the spinal cord indicates that neurons attempt to activate this neuroprotective system. However, the functionality depends on the expression of the ligand. Here, we performed RNAscope for *il4* and *il13* on spinal cords of healthy and EAE mice during disease peak. *Il4* mRNA expression was found in *vgat* + neurons of the spinal cord and was significantly increased in neurons from EAE mice in comparison to healthy mice (Fig. 8a-c). Moreover, we found *il13* mRNA expression markedly increased in *vgat* + spinal cord neurons of EAE peak mice (Fig. 8d-f), in line with reports of *il-13* upregulation in the cortex during traumatic brain injury [14]. *Il4* expression was observed throughout the neuronal cytoplasm (Fig. 8b´), whereas *il13* mRNA was in addition strongly expressed within the cell nucleus (Fig. 8e´), suggesting the presence of *il13* pre-mRNA in the neuroinflammatory state. These differences in the expression pattern and intensity of *il4* and *il13* in the EAE spinal cord may explain why IL-4 was shown to induce neuroprotective effects and promote axonal growth, whereas IL-13 did not [7].

We previously demonstrated beneficial effects of IL-4 in the EAE model, via neuronal IL-4R signaling, leading to outgrowth and repair [7, 8]. In addition, our recent study in healthy animals showed a role for IL-4 in fine-tuning of synaptic transmission [9]. Both homeostatic and therapeutic effects were abolished in IL-4Rα-deficient mice. The widespread CNS expression of IL-4Rα, IL-13Rα1, and IL-2Rγ, as well as the corresponding ligands, suggests a role for the IL-4R system in many brain functions.

## Conclusions

To date, only sparse information exists on neuronal expression of IL-4Rα in the CNS, and even less is known about the receptor co-chains IL-13Rα1 and IL2Rγ. This lack of detail stands in sharp contrast to the known functional effects of exogenously applied IL-4 in various CNS disease models, including neuroinflammation, and to the synaptic effects during homeostasis. Here, we show that neurons in the healthy brain express IL-4R subtypes, predominantly IL-4R type II, in various brain regions. IL-4 and IL-13 levels are low in healthy mice during homeostasis. The differential expression of the IL-4R subtypes in the spinal cord strongly indicates that the receptor is differentially regulated within the CNS. During neuroinflammation, IL-4Rα, IL-4, and IL-13 are elevated in neurons, indicating an autocrine upregulation of both ligands and receptor. We conclude that immune-challenged neurons upregulate both type 2 cytokines and IL-4Rα and speculate that these upregulations represent a neuronal attempt to activate mechanisms promoting neuroprotection and -regeneration.

## Supplementary Information

Below is the link to the electronic supplementary material.

Supplementary Material 1

## Data Availability

Data that support the findings of this study are available from the corresponding author upon request.

## Abbreviations

BLA: Basolateral amygdala
CNS: Central nervous system
CD4: Cluster of differentiation 4
CamKII: Calcium/calmodulin-dependent protein kinase II
ChAT: Choline acetyltransferase
DAT: Dopamine transporter
dHx: Dorsal hippocampus
EAE: Experimental autoimmune encephalomyelitis
FRA: Frontal association cortex
IFNγ: Interferon gamma
IL: Interleukin
IL-13Rα1 / il13ra1: Interleukin-13 receptor alpha one
IL-2Rγ / il2rg: Interleukin-2 receptor gamma
IL-4Rα / il4ra: Interleukin-4 receptor alpha
MC: Motor cortex
MOG: Myelin oligodendrocyte protein
PFC: Prefrontal cortex
nefh: Neurofilament h
NeuN: Neuronal nuclear protein N
rps26: Ribosomal protein S26
TNFα: Tumor necrosis factor alpha
vgat: Vesicular GABA transporter
vglut: Vesicular glutamate transporter
