# Supplementary material for: Neurons differentially upregulate type 2 immune cytokines and interleukin-4 receptor subtypes during neuroinflammation
Source: Acta Neuropathol Commun. 2026 Feb 12;14:74. doi: 10.1186/s40478-026-02229-7 (PMC13040743; doi:10.1186/s40478-026-02229-7)
Supplement: Supplementary file 1 — Supplementary Material 1 [file 40478_2026_2229_MOESM1_ESM.docx]

**SUPPLEMENTAL FIGURES**


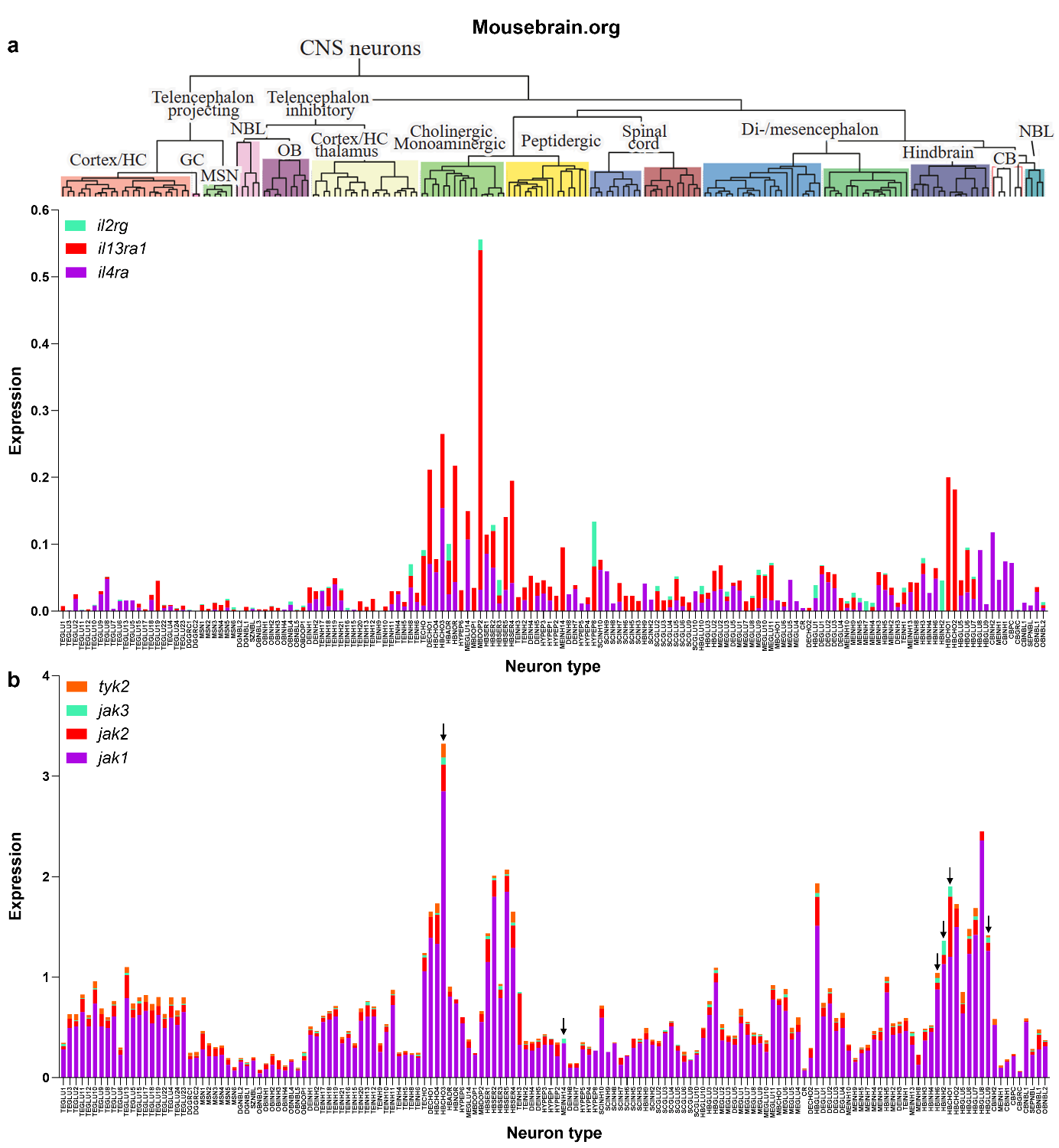


**Supplemental figure 1: *il4r* chain expression in CNS neurons.** Expression data extracted from the published mouse brain scRNAseq database (mousebrain.org [17])**. a** Expression of *il4ra*, *il2rg*, and *il13ra1* in different neuron types (employing expression level threshold of 0.001). **b** Expression of *jak1-3* and *tyk2* in the same set of neurons. *Jak 1*, and *-2* are most abundant, whereas *jak3* is hardly expressed in the forebrain compared to more distal neurons (arrows).


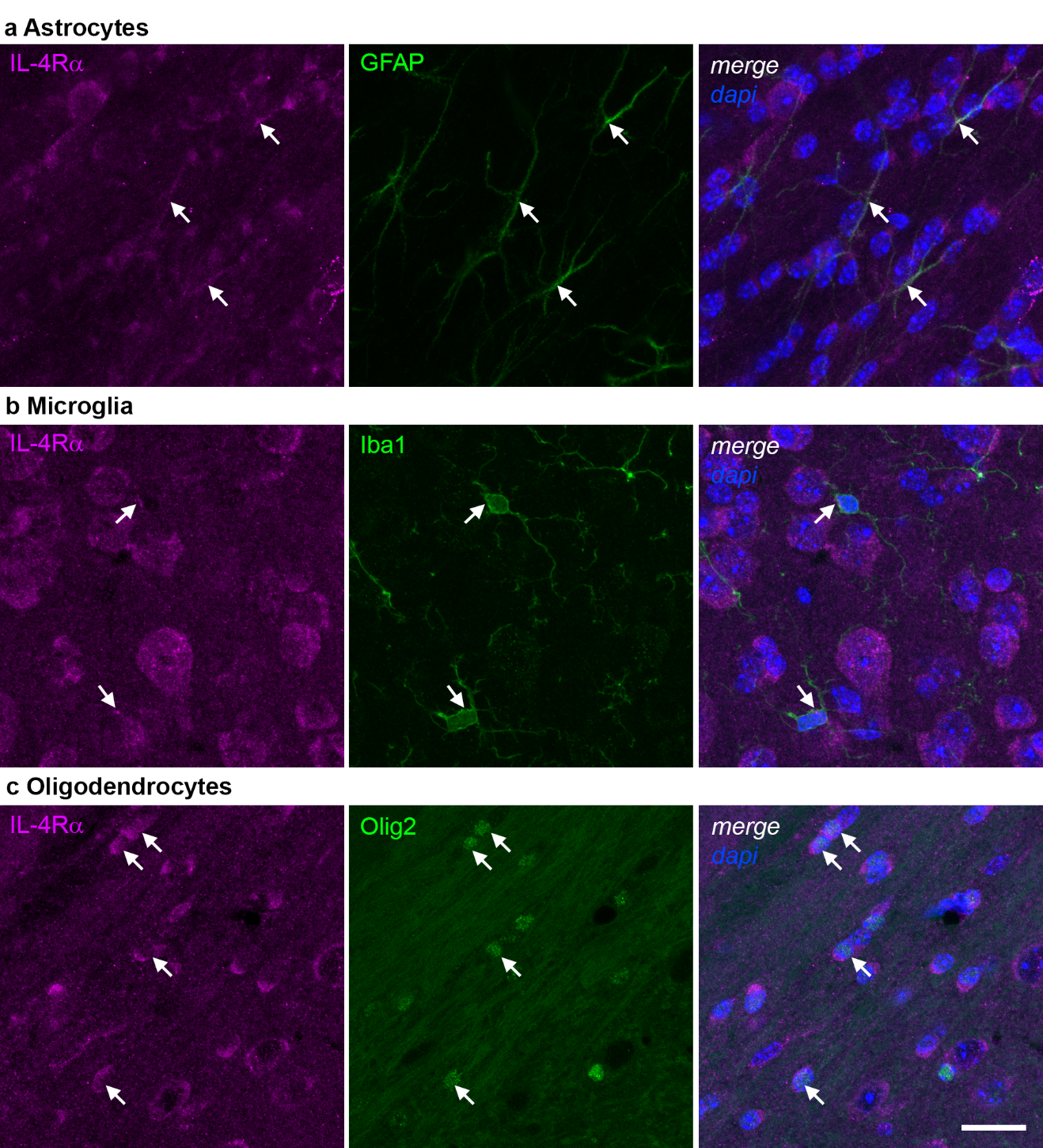


**Supplemental figure 2: Expression of IL-4Rα in glia.** Immunohistochemistry for IL-4Rα (magenta), dapi, and the markers **a** glial fibrillary acidic protein (GFAP) for astrocytes, **b** ionized calcium-adapter molecule 1 (Iba1) for microglia, and **c** oligodendrocyte transcription factor 2 (Olig2) for oligodendrocytes. Arrows point to cells expressing IL-4Rα and the respective glial cell marker. Scale bar = 20 µm.


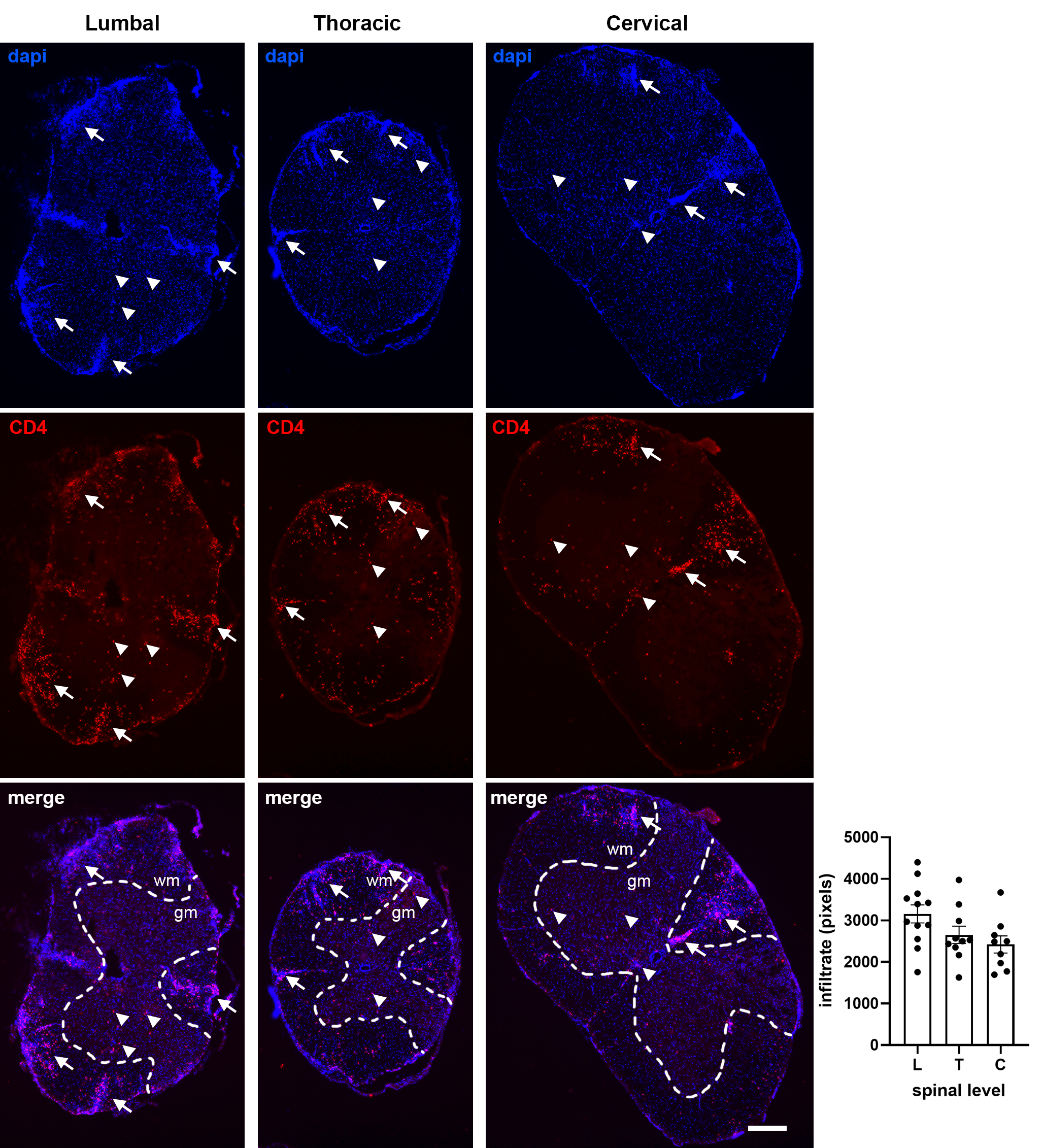


**Supplemental figure 3: Immune cell infiltration in spinal cord at EAE peak.** Immunohistochemistry for CD4 (red) and dapi (blue) in spinal cord of EAE peak mice at different spinal levels. No difference in infiltrate abundance between spinal levels was observed. Larger infiltrates (arrows) were observed in the wm, in contrast to some sparse CD4^+^ cells (arrowheads) in the gm. Scale bar = 200 µm. Abbreviations: gm = grey matter, wm = white matter.


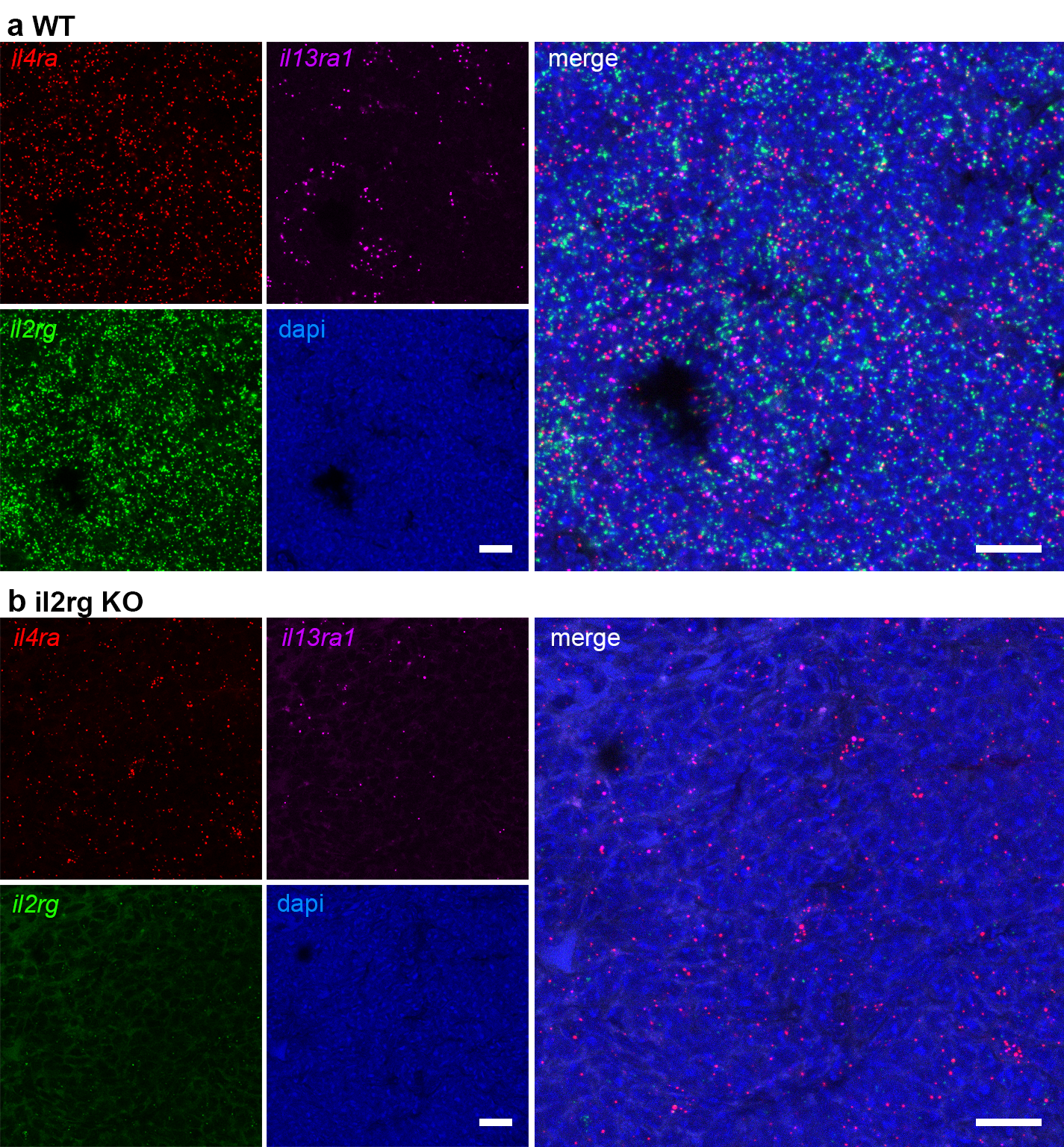


**Supplemental figure 4: Receptor chain expression in spleen.** RNAscope for *il4ra* (red), *il13ra1* (magenta), *il2rg* (green), with dapi (blue), in spleen sections from **a** healthy and **b** il2rg KO mice. Scale bars = 50 µm.


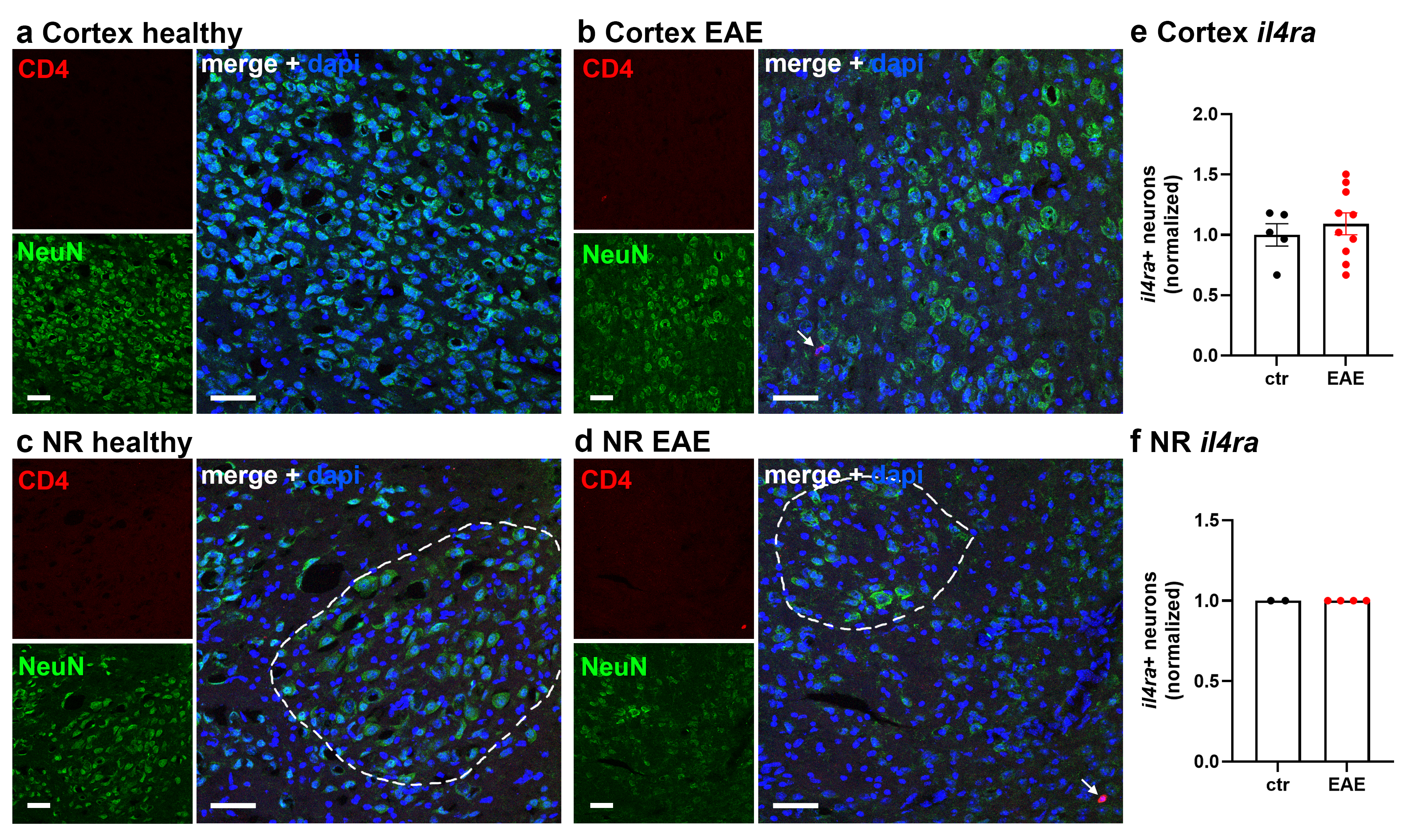


**Supplemental figure 5: Immune cell infiltration in cortex and NR at EAE peak.** Immunohistochemistry for CD4 (red, arrows point to sparse immune cells), NeuN (green), and dapi (blue) in **a-b** cortex and NR (dashed outline) **c-d** of healthy and EAE peak mice. Scale bar = 50 µm. Quantification of neurons expressing *il4ra* (for images see fig. 7) normalized to control in **e** cortex, and **f** NR revealed no significant difference between EAE and healthy controls.


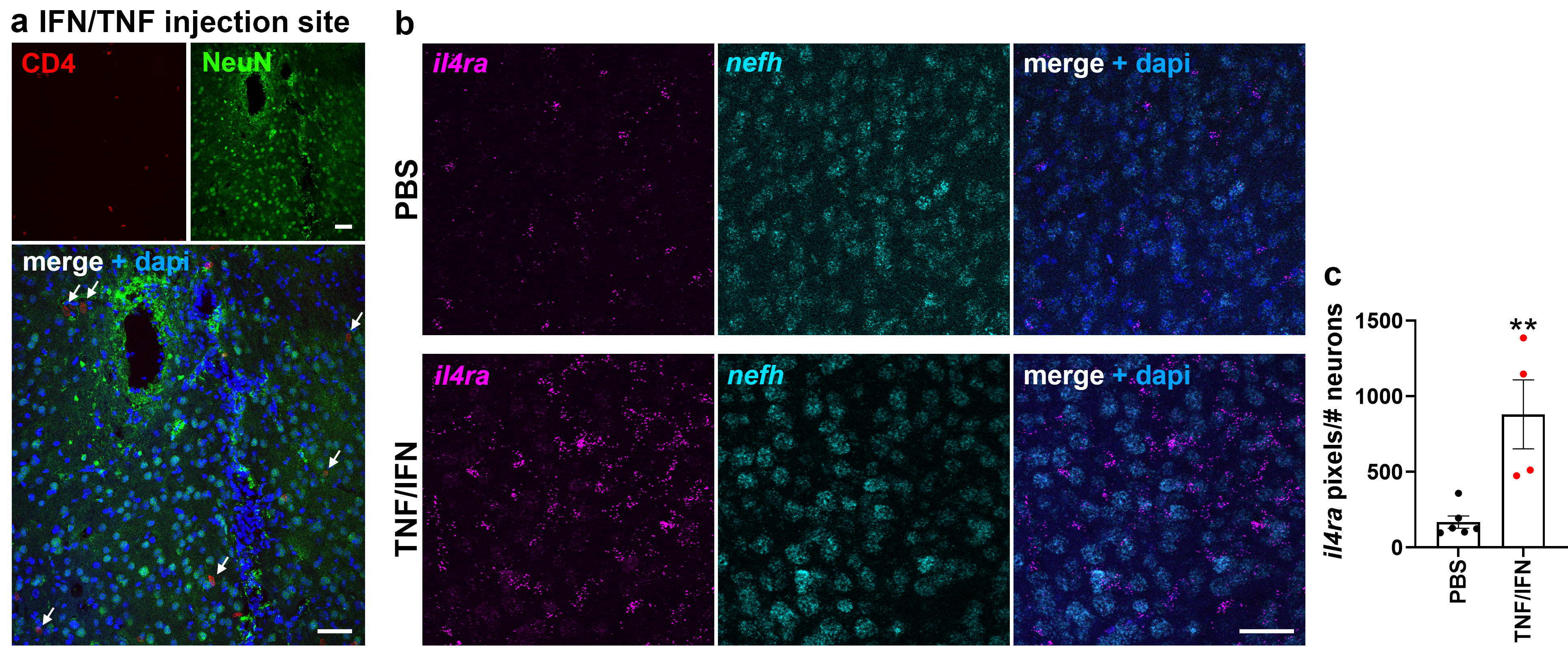


**Supplemental figure 6: Proinflammatory cytokine injection in the brain induces *il4ra* mRNA. a** Immunohistochemistry for CD4 (red), NeuN (green), with dapi (blue) at the site of IFN/TNF injection in the cortex, showing CD4^+^ cell infiltration (arrows) close to the damaged parenchyma. **b** RNAscope images for *il4ra* (magenta), *nefh* (cyan), with dapi (blue), in the motor cortex near the injection site, 2 d after PBS (upper panels) or IFN/TNF (lower panels) injection. **c** Quantification of the *il4ra* signals normalized to the number of neurons (n = 2-3 sections from 2 animals per group). Scale bars = 50 µm. Statistical analysis: unpaired T-test, ** p < 0.01.
